# Tumeur extra-durale rachidienne chez un enfant à l’hôpital universitaire de Constantine (Algérie): quel est votre diagnostic?

**DOI:** 10.48327/mtsi.v5i4.2025.710

**Published:** 2025-12-02

**Authors:** Mohamdi NABIL, Badreddine ALLOUACHE

**Affiliations:** 1. Département de médecine, Université de Batna 2, Laboratoire de parasitologie-mycologie, Centre hospitalier universitaire de Batna, 05000 Batna, Algérie; 2. Département de médecine, Université de Constantine, Laboratoire de parasitologie-mycologie, Centre hospitalier universitaire de Constantine, Constantine, Algérie

**Keywords:** Paraparésie spastique bilatérale, Hyporéflexie ostéotendineuse aux membres inférieurs, Signe de Babinski bilatéral, Troubles sphinctériens à type de mictions impérieuses, Constantine, Algérie, Afrique du Nord, Bilateral spastic paraparesis, Osteotendinous hyporeflexia in the lower limbs, Bilateral Babinski sign, Sphincter disorders such as urinary urgency, Constantine, Algeria, North Africa

## Abstract

Un enfant de 8 ans, originaire de la région de Constantine, sans antécédents pathologiques particuliers a été admis pour une faiblesse progressive des membres inférieurs évoluant depuis une vingtaine de jours, associée à des troubles de la marche.

Quel est votre diagnostic?

## Abstract (en)

A eight-year-ol d child from the Constantine region with no significant medical history was admitted due to progressive weakness in the lower limbs over a period of approximately 20 days, accompanied by difficulty walking.

What is your diagnosis?

## Observation

Un enfant de 8 ans, originaire de la région de Constantine, sans antécédents pathologiques particuliers a été admis pour une faiblesse progressive des membres inférieurs évoluant depuis une vingtaine de jours, associée à des troubles de la marche.

L’examen clinique initial, réalisé au service de neurologie du CHU de Constantine, a mis en évidence:

- une paraparésie spastique bilatérale (force musculaire cotée à 3/5);
- une hyporéflexie ostéotendineuse aux membres inférieurs;
- un signe de Babinski bilatéral;
- des troubles sphinctériens à type de mictions impérieuses.

La radiographie standard du rachis dorsal était normale.

Les examens biologiques réalisés à l’admission ont retrouvé:

- une numération formule sanguine (NFS), une CRP et une glycémie normales;
- une sérologie hydatique négative, réalisée par la technique d’hémagglutination indirecte.

**Figure 1:**
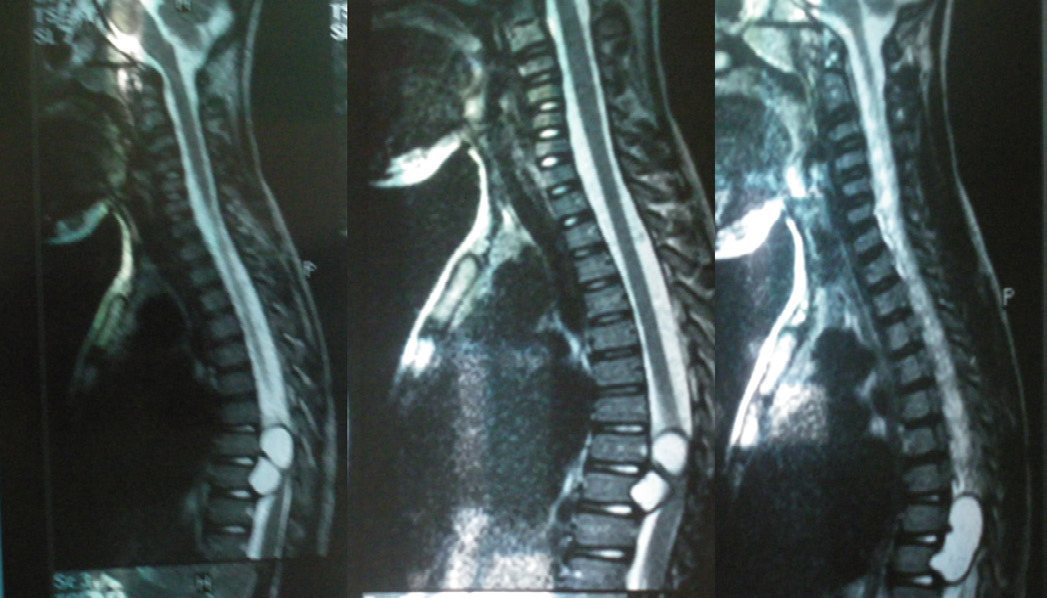
IRM médullaire dorsale

Une imagerie par résonance magnétique (IRM) médullaire dorsale a été réalisée. Elle a objectivé:

- un processus kystique extra-dural, latéralisé à gauche;
- s’étendant de D8 à D9;
- refoulant le sac dural vers la droite;
- sans anomalie osseuse associée (Fig. 1).

Quel est votre diagnostic?

Les hypothèses diagnostiques envisagées étaient les suivantes:

- kyste arachnoïdien extra-dural,
- tumeur nerveuse bénigne (neurinome, schwannome),
- abcès épidural froid tuberculeux,
- lymphome épidural,
- lésion parasitaire kystique (dont kyste hydatique).

Le patient a bénéficié, dans les 48 heures suivant l’admission, d’une laminectomie D8-D9, au bloc opératoire de neurochirurgie sous anesthésie générale.

L’intervention a permis l’exérèse complète d’un kyste à paroi fine contenant un liquide clair, situé entre l’arc postérieur de D8 et la lame gauche de D9, refoulant le sac dural vers la droite.

**Figure 2:**
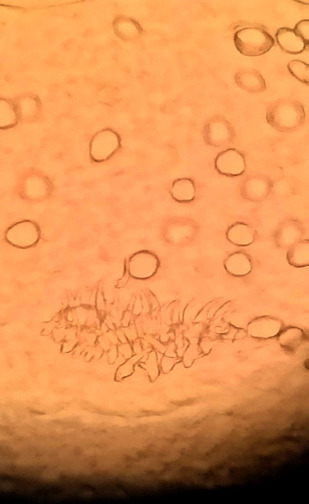
Observation à l’objectif X40 de la pièce opératoire

La pièce opératoire, acheminée au laboratoire, a révélé la présence de scolex et de crochets caractéristiques d’un parasite du genre *Echinococcus* (Fig. 2), confirmant le diagnostic de kyste hydatique extra-dural rachidien isolé.

Les troubles sphinctériens se sont résorbés au bout de deux semaines de rééducation. Le traitement post-opératoire comprenait une antibioprophylaxie et une chimiothérapie antiparasitaire par albendazole (10 mg/kg/j) pendant 3 mois, associées à des séances de kinésithérapie motrice. L’évolution clinique du patient a été progressivement favorable. La force musculaire a été retrouvée dès les premières semaines, avec un passage de 3/5 à 4/5 aux deux membres inférieurs. Un suivi par IRM à 6 mois avait été programmé pour rechercher une éventuelle récidive mais le patient a été perdu de vue après sa sortie.

## Discussion

L’association d’un syndrome de compression médullaire subaigu chez un enfant, sans contexte infectieux ni antécédents notables et la découverte à l’IRM d’un processus kystique extra-dural bien circonscrit, sans atteinte osseuse, orientent vers plusieurs hypothèses diagnostiques dont certaines ont été évoquées dans notre cas avant la mise en évidence de scolex dans la pièce opératoire [3]. Les arguments épidémiologiques en faveur d’une échinococcose kystique étaient:

- enfant vivant en milieu rural dans une région d’endémie parasitaire;
- profession paternelle: berger, avec contact possible avec des chiens et des ovins.

Les arguments apportés par l’imagerie étaient:

- masse kystique extra-durale postérieure, de signal homogène, sans prise de contraste, refoulant le sac dural;
- absence de lyse osseuse ou d’atteinte vertébrale;
- imagerie compatible avec une lésion compressive bénigne à développement lent.

Et les arguments biologiques étaient:

- bilan inflammatoire normal;
- absence de syndrome infectieux ou tumoral.

La présence de scolex et de crochets d’E. *granulosus* dans la pièce opératoire a permis de s’assurer que l’identification spécifique a bien été faite.

Le test d’hémagglutination indirecte était négatif, ce qui n’est pas surprenant car une sérologie négative n’exclut pas une hydatidose, notamment pour les localisations osseuses ou rachidiennes. Dans la littérature, il est mentionné que la sensibilité de la sérologie est fonction du stade évolutif de la maladie et du type de localisation; au cours des localisations osseuses la sérologie ne serait positive que dans 25 à 56% des cas [2,3].

Plusieurs auteurs ont observé des résultats faussement négatifs dans ces localisations, Cela s’explique par une libération antigénique limitée, notamment lorsque les kystes sont anciens, calcifiés ou confinés dans l’os. Par exemple, dans l’étude de Song *et al.,* 2007, il est indiqué que les tests sérologiques sont fréquemment négatifs quand le kyste est âgé ou calcifié [4].

Dans notre cas, l’imagerie et surtout l’examen histologique ont permis de retenir le diagnostic, illustrant que la sérologie seule ne peut exclure l’hydatidose dans les localisations rachidiennes. Le kyste hydatique extra-dural constitue une forme rare de l’hydatidose osseuse, représentant moins de 2% des localisations, avec une prédominance des atteintes vertébrales. La forme intrarachidienne primitive, sans atteinte osseuse, est exceptionnelle, surtout chez l’enfant. Dans ces cas, la colonne dorsale est le site le plus fréquent, suivie du rachis lombaire [1, 2-3].

La dissémination du parasite se fait probablement par voie hématogène, *via* les plexus veineux paravertébraux, expliquant sa prédilection pour l’espace épidural richement vascularisé [2,3]. La croissance lente du kyste entraîne une compression médullaire progressive, souvent révélée par un syndrome pyramidal avec paraparésie, hyperréflexie et troubles sphinctériens [2,3].

L’IRM est l’examen de choix, montrant une masse kystique bien limitée, hyperintense en T2, sans prise de contraste, typique mais non spécifique [1,2]. La sérologie peut être négative dans les formes osseuses, ce qui rend l’examen parasitologique direct sur pièce opératoire indispensable au diagnostic [1,2].

Le traitement repose sur une exérèse chirurgicale complète, sans rupture, complétée par une chimiothérapie antiparasitaire à base d’alben-dazole pendant 2 à 3 mois [1,2]. Le pronostic neurologique est généralement favorable en cas de prise en charge précoce mais un suivi IRM à long terme est nécessaire pour dépister une récidive.

## Conclusion

Ce cas illustre l’importance de sensibiliser les praticiens en zones d’endémie aux présentations atypiques de l’hydatidose vertébrale, afin d’améliorer le délai diagnostique et le pronostic des patients atteints de cette pathologie rare. Il souligne également le manque de fiabilité de la sérologie qui peut, lorsqu’elle est négative, retarder la prise en charge d’une hydatidose.

## Financement

Cette étude n’a bénéficié d’aucun financement spécifique de la part d’organismes prblics, commerciaux ou à but non lucratif.

## Contributions des auteurs

Mohamdi NABIL: conception de l’étude, collecte des données cliniques, rédaction du manuscrit. Badreddine ALLOUACHE: analyse des données cliniques, suivi du patient.

Tous les auteurs ont relu, révisé et approuvé la version finale du manuscrit.

## Déclaration de liens d’intérêt

Aucun lien d’intérêt n’a été déclaré.

## References

[1] Brunetti E, Kern P, Vuitton DA (2010). ; Writing Panel for the WHO-IWGE. Expert consensus for the diagnosis and treatment of cystic and alveolar echinococcosis in humans. Acta Trop.

[2] Meng Y, Ren Q, Xiao J, Sun H, Huang Y, Liu Y, Wang S, Wang S (2023). Progress of research on the diagnosis and treatment of bone cystic echinococcosis. Front Microbiol.

[3] Neumayr A, Tamarozzi F, Goblirsch S, Blum J, Brunetti E (2013). Spinal cystic echinococcosis-a systematic analysis and review of the literature: part 1. Epidemiology and anatomy. PLoS Negl Trop Dis.

[4] Song XH, Ding LW, Wen H (2007). Bone hydatid disease. Postgrad Med J.

