# Supplementary material for: Extradural spinal tumor in a child at Constantine University Hospital (Algeria): what is your diagnosis?
Source: Med Trop Sante Int. 2025 Dec 2;5(4):mtsi.v5i4.2025.710. [Article in French] doi: 10.48327/mtsi.v5i4.2025.710 (PMC12948367; doi:10.48327/mtsi.v5i4.2025.710)
Supplement: Related texts

# Research article: Extradural spinal tumor in a child at Constantine University Hospital (Algeria): what is your diagnosis?

## Case observation

An eight-year-old child originating from the Constantine region with no significant medical history was admitted due to progressive weakness in the lower limbs over a period of approximately 20 days, accompanied by difficulty walking.

The initial clinical examination, carried out in the Neurology Department at Constantine University Hospital, revealed the following:

- Bilateral spastic paraparesis (muscle strength rated at 3/5);
- Osteotendinous hyporeflexia in the lower limbs;
- Bilateral Babinski sign;
- Sphincter disorders, such as urgent urination.

The standard X-ray of the dorsal spine was normal. Laboratory tests performed upon admission revealed:

- Normal complete blood count (CBC), C-reactive protein (CRP), and blood glucose levels;
- Negative hydatid serology using the indirect hemagglutination technique.

A spinal magnetic resonance imaging (MRI) revealed :

- An extradural cystic process lateralized to the left;
- Extending from D8 to D9;
- Displacing the dural sac to the right;
- With no associated bone abnormalities (Fig. 1).

**Figure 1:** Spinal MRI

What is your diagnosis?

The following diagnostic hypotheses were considered:

- Extradural arachnoid cyst;
- Benign nerve tumor (neurinoma or schwannoma);
- Tuberculous cold epidural abscess;
- Epidural lymphoma;
- Cystic parasitic lesion (including hydatid cyst).

Within 48 hours of admission, the patient underwent a D8-D9 laminectomy in the neurosurgery operating room under general anesthesia.

The procedure allowed for the complete removal of a thin-walled cyst containing clear fluid. The cyst was located between the posterior arch of D8 and the left lamina of D9 and was pushing the dural sac to the right.

The surgical specimen sent to the laboratory revealed scolex and hooks characteristic of a parasite of the genus *Echinococcus* (Fig. 2), confirming the diagnosis of an isolated extradural spinal hydatid cyst.

**Figure 2:** Observation of the surgical site at 40x magnification

The sphincter disorders resolved after two weeks of rehabilitation. Postoperative treatment included antibiotic prophylaxis and antiparasitic chemotherapy with albendazole (10 mg/kg/day) for three months, combined with physical therapy sessions. The patient’s clinical condition gradually improved. Muscle strength increased from 3/5 to 4/5 in both lower limbs within the first few weeks. A follow-up MRI had been scheduled at six months to check for possible recurrence, but the patient was lost to follow-up after discharge.

## Discussion

The combination of subacute spinal cord compression syndrome in a child with no infectious context or notable history and the discovery of a well-circumscribed extradural cystic process with no bone involvement on MRI points to several diagnostic hypotheses. Some of these hypotheses were considered in our case before scolex were discovered in the surgical specimen [3].

The epidemiological arguments in favor of cystic echinococcosis were:

- The child lived in a rural area in an endemic region;
- The father was a shepherd with possible contact with dogs and sheep.

The imaging-based arguments were:

- Posterior extradural cystic mass with homogeneous signal and no contrast uptake and displacing the dural sac;
- Absence of bone lysis or vertebral involvement;
- Imaging consistent with a slow-growing benign compressive lesion;

The biological arguments were:

- A normal inflammatory profile;
- An absence of infectious or tumor syndrome.

The presence of scolex and hooks of *E. granulosus* in the surgical specimen confirmed that the specific identification had been made correctly The indirect hemagglutination test was negative, which is not surprising, as negative serology does not rule out hydatid disease, particularly in bone or spinal locations. The literature mentions that serology sensitivity depends on the disease stage and location. In bone locations, serology is positive in only 25 to 56% of cases [2,3].

Several authors have observed false-negative results in these locations. This can be explained by limited antigen release, particularly when the cysts are old, calcified, or confined to the bone. For example, Song *et al.* (2007) indicate that serological tests are frequently negative when the cyst is old or calcified [4].

In our case, the diagnosis was made through imaging and, most importantly, histological examination, which illustrates that serology alone cannot rule out hydatid disease in spinal locations. Extradural hydatid cysts are a rare form of bone hydatid disease, accounting for less than two percent of cases, predominantly involving the vertebrae. The primary intraspinal form without bone involvement is exceptional, particularly in children. In these cases, the thoracic spine is the most common site, followed by the lumbar spine [1, 2-3].

The parasite likely spreads hematogenously via the paravertebral venous plexuses, which explains its predilection for the richly vascularized epidural space [2,3]. The cyst’s slow growth leads to progressive spinal cord compression, which is often revealed by a pyramidal syndrome with paraparesis, hyperreflexia, and sphincter disorders [2,3]. MRI is the examination of choice, showing a well-defined cystic mass that is hyperintense on T2 and does not enhance with contrast. This finding is typical but not specific [1,2]. Serology may be negative in bone forms, so direct parasitological examination of the surgical specimen is essential for diagnosis [1,2].

Treatment involves complete surgical excision without rupture, supplemented by 2-3 months of antiparasitic chemotherapy with albendazole [1,2]. The neurological prognosis is generally favorable with early surgical and medical treatments; however, long-term MRI follow-up is necessary to detect recurrence.

## Conclusion

This case highlights the importance of raising awareness among practitioners in endemic areas about the atypical presentations of vertebral hydatid disease. This will improve diagnostic accuracy and prognosis for patients with this rare condition. It also underscores the unreliability of serology whose negative results can delay the management of hydatid disease.

## Funding

This study did not receive any specific funding from public, commercial, or nonprofit organizations.

## Authors’ contributions

Mohamdi NABIL: Study design, clinical data collection, and manuscript writing.

Badreddine ALLOUACHE: Clinical data analysis and patient follow-up.

All authors reviewed, revised, and approved the final version of the manuscript.

## Conflicts of interest

The authors declare no conflicts of interest.
